# The economic burden of Hospital-Acquired Infections on cancer patients under China’s pioneering Diagnosis-Intervention Packet payment model: a retrospective study

**DOI:** 10.3389/fpubh.2026.1798805

**Published:** 2026-04-16

**Authors:** Jiao Liu, Min Xu, Xu Ju, Xue-er Peng, Xue Xia, Huan Li

**Affiliations:** 1 Department of Hospital Infection Control, State Key Laboratory of Oncology in South China, Guangdong Key Laboratory of Nasopharyngeal Carcinoma Diagnosis and Therapy, Guangdong Provincial Clinical Research Center for Cancer, Sun Yat-sen University Cancer Center, Guangzhou, China; 2 Medical Insurance Office, State Key Laboratory of Oncology in South China, Guangdong Key Laboratory of Nasopharyngeal Carcinoma Diagnosis and Therapy, Guangdong Provincial Clinical Research Center for Cancer, Sun Yat-sen University Cancer Center, Guangzhou, China

**Keywords:** cancer, diagnosis-intervention packet, economic burden, medical insurance payment reform, nosocomial infection, public health

## Abstract

**Background:**

This study aimed to analyze the distribution and economic burden of cancer-related nosocomial infections under the Diagnosis-Intervention Packet (DIP) payment model.

**Methods:**

A retrospective study was conducted using 2024 medical insurance and healthcare-associated infection (HAI) data from discharged patients at a regional cancer center. We compared the differences in medical insurance settlement indicators between HAI and non-HAI patients within major DIP categories, as well as among different HAI types. After adjusting for time-dependent bias and confounding factors via risk-set matching, we further compared the above indicators between HAI and non-HAI patients, and generalized linear model (GLM) was used to identify independent factors for DIP settlement difference.

**Results:**

HAIs were most concentrated in the DIP category *Malignant neoplasm of thoracic esophagus: Partial esophagectomy via cervical-thoracic-abdominal three-incision approach*. With major DIP categories, the infected group had significantly higher hospitalization costs, diagnostic test and procedure costs, longer length of stay, and greater DIP deficits (all *p <* 0.05). MDRO type count and surgical site wound contamination grade were positively correlated with the above indicators and DIP deficits (all *p* < 0.05). After risk-set matching adjustment, there were still differences between the infected and non-infected groups in the above medical insurance settlement indicators (all *p <* 0.05). HAIs led to an increase of $5,031.01 in hospitalization costs, $798.81 in diagnostic test and procedure costs, 9 days in prolonged hospital stay, and an additional $1,436.27 in DIP deficits. Generalized linear model analysis showed that HAI occurrence and the number of MDRO types were independent risk factors affecting DIP payment differentials (all *p <* 0.05). HAI occurrence and infection with ≥3 MDRO types led to an increase of $2,021.15 and $5,601.44 in DIP deficits, respectively.

**Conclusion:**

Using DIP payment differentials to assess the economic burden of HAIs is more scientifically valid. Cancer-associated HAIs significantly increase medical resource consumption and economic burden. Implementing infection prevention and control strategies at the DIP categories, particularly for MDRO infections, may help reduce additional costs and improve the balance of medical insurance payments.

## 1 Introduction

The sustainability of medical security and equitable health for all are core concerns in the field of global public health, as well as critical issues in reform and development. The Fee-for-Service (FFS) mode, which tends to induce excessive medical practices, has been recognized as a major driver of healthcare costs (1). With the deepening reform of China’s medical insurance payment system reform, Diagnosis-Related Groups (DRG) and Diagnosis-Intervention Packet (DIP) have gradually replaced the traditional project-based payment mode since 2019, becoming core mechanisms for optimizing medical resource allocation (2–4). Although DRG remains the predominant payment method in developed countries, its limitations in assessing resource consumption homogeneity led Chinese researchers to develop the DIP methodology. This innovative approach leverages clinical big data to objectively classify medical records objectively based on “principal diagnosis + primary procedure” combinations, grouping cases with shared clinical characteristics and resource utilization patterns (5). Multiple studies have confirmed that DIP significantly enhances hospitals’ cost containment capabilities and reduces healthcare expenditures, leading to its rapid expansion across China. Currently, DIP implementation covers twice as many cities as DRG pilot sites (6, 7).

Within the context of healthcare reform, the economic impact of Healthcare-Associated Infections (HAIs) has been substantially amplified (8). Cancer patients represent a critically vulnerable population, for whom infections constitute the most frequent complication and the second leading cause of mortality (9, 10). Factors such as chemotherapy-induced neutropenia, immunosuppression, and increased invasive procedures elevate their infection-related fatality risk to three times that of non-oncological patients (11). Under the DIP payment mode, cancer-related hospital infections not only exacerbate resource overutilization but also directly affect the efficiency of disease score settlement. However, the academic community has not yet clearly quantified the precise economic burden of healthcare-associated infections (HAIs) in cancer patients under the Diagnosis-Intervention Packet (DIP) payment scenario, especially the scale of medical insurance fund loss. This makes it difficult to support the solution to the core public health problem of synergy between medical insurance payment reform and infection control.

Therefore, this study focuses on the economic burden of HAIs among cancer patients under the DIP payment system. By quantifying the impact of HAIs and related risk factors on medical insurance settlement indicators, we describe the additional economic burden on healthcare funds and its variation across subgroups. The findings may provide useful evidence for integrating infection control considerations into hospital management under the DIP framework.

## 2 Materials and methods

### 2.1 Study design and participants

This retrospective cohort study analyzed all hospital discharges from a large regional cancer center in southern China between January and December 2024. Patients with community-acquired infections at admission were excluded. Overall, study subjects were divided into two groups: (1) Infected group, defined as patients who developed HAIs during hospitalization; (2) Non-infected group, defined as patients without any infection during hospitalization.

Medical insurance data were extracted from the local DIP Integrated Management System, and HAI data were obtained from the Nosocomial Infection Manage System of the center. Primary outcome measures included HAI incidence rate, hospitalization costs, length of stay, diagnostic tests and procedures, DIP grouping, and DIP payment differentials. All cost data were converted to US dollars based on the 2024 annual average exchange rate (USD 1 = CNY 7.12).

### 2.2 Definitions

HAIs were diagnosed according to the *Diagnostic Criteria for Healthcare-Associated Infections (Trial Implementation)* established by the original Ministry of Health of the People’s Republic of China (12), which was developed with reference to the criteria from the US Centers for Disease Control and Prevention (13). Infections occurring ≥48 h after admission were identified and reported jointly by infection prevention and control professionals and attending clinicians.

HAI incidence rate refers to the proportion of newly developed Hospital-Acquired Infections (cases) among hospitalized patients within a specified time period. Calculated as: HAI incidence rate = Number of newly diagnosed hospital-acquired infection cases during the specified period/Total number of hospitalized patients during the same period ×100%.

DIP payment differentials were defined as the difference between the standard DIP payment for a specific diagnosis-intervention group and the actual hospitalization cost. Calculated as: DIP payment differentials = DIP Standard Payment - Actual Cost. Positive value indicates a surplus, negative value indicates a hospital loss. DIP payment differentials were calculated for all discharged patients.

Surgical wound classification followed the *Instructions for Completing Specific Sections of the Inpatient Basic Data Sheet* issued by the National Health Commission of China (14). Category 0 incision refers to surgical procedures without a skin incision or with a laparoscopic incision; Category I: sterile incision; Category II: contaminated incision; Category III: infected incision.

### 2.3 Matching design

To minimize confounding factors and time bias, our study employed 3 models to compare differences between infected and non-infected groups. Only successfully matched pairs were included in the subsequent analyses.

Model 1: conventional 1:1 exact matching was performed to match HAI cases with non-HAI controls, based on (1) identical Diagnosis-Intervention Packet (DIP) groups according to the DIP Repository (Version 2.0) (15); (2) gender; (3) age difference within 5 years; (4) Charlson age comorbidity index (CACI) (16, 17). Cases and controls were matched within the same CACI risk stratum (high-risk: CACI > 3; low-risk: CACI ≤ 3). The CACI score was assigned based on comorbidities corresponding to the International Classification of Diseases, 10th Revision (ICD-10) codes in patients’ diagnoses (Supplementary Table S1).

Model 2: Propensity score matching (PSM) was conducted using a 1:1 nearest-neighbor method with a caliper width of 0.2, adopting the same matching covariates as Model 1 to adjust for intergroup confounding bias.

Model 3: Risk-set matching (the primary analytical model) was applied to adjust for time-dependent bias (18). Each HAI case infected at time T1 is matched to a patient not yet infected at time T1 rather than a never-infected patient, and 1:1 matching was implemented using the same matching variables as Model 1.

### 2.4 Statistical analysis

The data were analyzed using R software (Version 4.3.1) and IBM SPSS Statistics 31.0 (IBM Corp., Armonk, NY, USA). Continuous variables presented as median with interquartile range [M (P25, P75)] due to non-normal distribution, were compared using the Mann–Whitney U test (two groups) or Kruskal–Wallis test (multiple groups). Point estimates and 95% confidence intervals (95% CI) for median differences (MD) were calculated via the Hodges-Lehmann estimation method. Categorical variables were expressed as counts (percentage) [*n* (%)], with group differences assessed by *χ*^2^ test.

Based on the risk-set matching samples, generalized linear models with Gaussian distribution and robust identity link function were constructed to explore the influencing factors of DIP payment differentials. A two-sided *α* level of 0.05 was used, with statistical significance defined as *p* < 0.05.

## 3 Results

### 3.1 Basic information

A total of 205,569 inpatient cases were included in the study, of which 122,725 (59.70%) were from the pilot region and included in DIP settlement (DIP group), while 82,844 (40.30%) were not included in DIP settlement (non-DIP group). Among the discharged patients, 803 cases (0.39%) of Hospital-Acquired Infections occurred, including 490 cases (0.40%) from the DIP group and 312 cases (0.38%) from the non-DIP group. The infection rates did not differ significantly between the two groups (*p* > 0.05) (Supplementary Table S2).

### 3.2 Medical insurance settlement indicators for major DIP categories

The top five DIP categories with the highest concentration of HAIs (hereafter referred to as “major DIP diseases”) are listed in Table 1. Among them, *Malignant neoplasm of thoracic esophagus: Partial esophagectomy via cervical-thoracic-abdominal three-incision approach* had the highest number of HAIs, with an incidence rate of 8.60%, accounting for 4.22% of all infections.

**Table 1:** Top 5 HAI incidence diseases: impact analysis on medical insurance payment indicators.

DIP categories	Total number of cases	Number of HAI cases	HAI incidence rate (%)	Infection percentage (%)	Variable	Infected group M(P25, P75)	Non-infected group M (P25, P75)	Median difference	95% CI	*p*-value
Malignant neoplasm of thoracic esophagus: partial esophagectomy via cervical-thoracic-abdominal three-incision approach	395	34	8.61	4.24	Hospitalization costs	25155.87 (18214.94, 36242.96)	16202.97 (13915.28, 19539.27)	9812.00	6763.00, 12921.00	<0.001
					Length of stay	31.00 (19.75, 48.00)	15.00 (12.00, 19.00)	15.00	10.00, 20.00	<0.001
					Diagnostic tests and procedures	4064.21 (2393.89, 6448.53)	1781.81 (1274.71, 2538.86)	2247.00	1489.00, 3118.00	<0.001
					DIP payment differentials	−731.36 (−7983.29, 905.91)	2837.45 (570.60, 4437.24)	−4667.50	−6063.00, −3264.00	<0.001
Malignant neoplasm of stomach: surgical procedure	746	29	3.89	3.62	Hospitalization costs	15114.32 (11017.31, 18237.89)	4286.04 (2938.71, 9154.92)	8938.00	7135.00, 10887.00	<0.001
					Length of stay	20.00 (15.00, 39.00)	9.00 (7.00, 13.00)	10.00	8.00, 14.00	<0.001
					Diagnostic tests and procedures	2860.53 (2314.06, 3717.86)	1512.47 (956.39, 1821.71)	1417.00	1126.00, 1737.00	<0.001
					DIP payment differentials	−1466.26 (−4447.96, 133.61)	11.12 (6.70, 894.85)	−1987.50	−3222.00, −1069.00	<0.001
Malignant neoplasm of rectum: laparoscopic anterior resection of rectum	452	20	4.42	2.49	Hospitalization costs	9970.29 (8235.74, 12435.42)	6559.29 (6029.50, 7813.53)	2791.50	1675.00, 3907.00	<0.001
					Length of stay	18.50 (15.00, 24.75)	10.00 (9.00, 13.75)	8.00	6.00, 10.00	<0.001
					Diagnostic tests and procedures	1589.87 (1397.62, 1974.07)	1130.90 (1000.31, 1428.21)	497.50	355.00, 681.00	<0.001
					DIP payment differentials	118.89 (−109.07, 1274.40)	2445.75 (1569.21, 2816.92)	−1660.50	−2175.00, −1023.00	<0.001
Malignant neoplasm of rectum: laparoscopic anterior resection of rectum with temporary ileostomy	286	19	6.64	2.37	Hospitalization costs	12546.12 (8422.33, 14308.06)	7713.985 (6531.40, 12459.05)	3785.00	1792.00, 5959.00	0.005
					Length of stay	15.00 (13.00, 21.00)	10.00 (9.00, 11.00)	6.00	4.00, 8.00	<0.001
					Diagnostic tests and procedures	1609.96 (1277.69, 2022.24)	1035.57 (867.27, 1167.61)	620.00	409.00, 850.00	<0.001
					DIP payment differentials	27.66 (−182.22, 2293.89)	2329.97 (30.23, 3565.52)	−2045.00	−3075.00, −738	0.008
Malignant neoplasm of cervix: radical hysterectomy with bilateral salpingo-oophorectomy	580	18	3.10	2.24	Hospitalization costs	16370.44 (7565.06, 22456.48)	5645.44 (5215.18, 6340.90)	10461.00	7424.00, 12689.00	<0.001
					Length of stay	18.00 (14.00, 24.00)	10.00 (9.00, 12.00)	16.00	10.00, 20.00	<0.001
					Diagnostic tests and procedures	2237.57 (1511.44, 3343.06)	1133.13 (930.98, 1536.60)	1014.00	625.00, 1465.00	<0.001
					DIP payment differentials	−2990.44 (−3303.58, −1087.53)	250.49 (−14.60, 552.34)	−3043.00	−3505.00, −2314.00	<0.001

*p*-values were estimated using Mann–Whitney U test. Median differences and 95% CIs were estimated using Hodges–Lehmann estimator.

All major DIP categories showed significantly higher hospitalization costs, diagnostic tests and procedures, and longer stays in the infected group versus the non-infected group (all *p* < 0.05), alongside lower DIP payment differentials in the infected group (*p* < 0.05) (Table 1). Three of the major DIP categories exhibited negative DIP payment differentials, indicating hospital losses. The greatest disparities in costs and length of stay were observed in *Malignant neoplasm of cervix: Radical hysterectomy with bilateral salpingo-oophorectomy*, where infected patients incurred $10,461 higher costs and 16 days longer stays. For DIP payment differentials and diagnostic tests and procedures, the largest inter-group difference was observed in *Malignant neoplasm of thoracic esophagus: Partial esophagectomy via cervical-thoracic-abdominal three-incision approach,* with a difference in deficit of $4,667.50 and a difference of $2,247 in diagnostic tests and procedures between the two groups.

### 3.3 Impact of different HAIs classifications on medical insurance settlement indicators

Both the frequency of HAIs and the diversity of MDROs during hospitalization were significantly associated with higher hospitalization costs, diagnostic tests and procedures, and length of stay (all *p* < 0.05). Although higher infection frequency showed a trend toward greater median DIP payment deficits, this association was not statistically significant, possibly due to limited sample sizes and variations in infection types within subgroups. In contrast, greater MDRO diversity was significantly associated with larger DIP payment deficits (*p* < 0.05) (Table 2).

**Table 2:** Impact of different HAI classifications on medical insurance settlement indicators.

Classification of HAIs	Hospitalization costs M (P25, P75)	*p*-value	Diagnostic tests and procedures M (P25, P75)	*p*-value	Length of stay M (P25, P75)	*p*-value	DIP payment differentials M (P25, P75)	*p*-value
Frequency of HAIs		<0.001^a^		<0.001^a^		<0.001^a^		0.052^a^
1 (*n* = 747)	12851.84 (8433.68, 18838.38)		2187.66 (1505.87, 3384.79)		21.00 (15.00, 29.00)		−563.28 (−2788.72, 41.28)	
2 (*n* = 50)	18607.59 (12332.55, 26508.42)		3936.13 (2539.86, 5382.22)		28.50 (20.75, 45.25)		−1376.67 (−3253.87, 20.92)	
3 (*n* = 5)	32282.47 (18096.79, 63940.77)		9615.91 (3552.40, 14702.29)		69.00 (50.50, 110.00)		−5528.52 (−7911.89, −777.65)	
Catheter-associated infections		0.012^b^		0.200^b^		0.237^b^		0.456^b^
Non^1^ (*n* = 756)	13313.69 (9126.77, 19567.05)		2304.64 (1557.85, 3500.97)		22.00 (15.00, 30.00)		−655.11 (−2841.50, 37.24)	
Yes (*n* = 46)	8648.24 (5577.55, 18733.06)		1936.33 (1037.80, 3599.07)		18.50 (12.75, 34.50)		−382.42 (1775.44, 45.20)	
Number of MDRO types involved^a^		<0.001^a^		<0.001^a^		<0.001^a^		<0.001^a^
0 (*n* = 598)	12854.32 (8528.00, 18983.58)		2275.94 (1541.58, 3455.91)		21.00 (14.00, 29.00)		−448.30 (−2634.46, 47.56)	
1 (*n* = 173)	13365.09 (8623.189, 18494.36)		2142.41 (1401.44, 3377.29)		22.00 (15.00, 31.00)		−993.85 (−2794.48, 24.908)	
2 (*n* = 19)	19907.25 (12412.16, 29865.57)		3575.49 (2040.07, 5220.89)		30.00 (20.00, 57.00)		−3289.63 (−5102.42, −973.05)	
≥3 (*n* = 12)	30839.017 (24169.327, 44573.50)		6002.94 (4927.57, 8076.37)		52.50 (33.00, 65.50)		−6512.35 (−8153.87, −3440.85)	
Surgical site infections		<0.001^a^		0.001^a^		<0.001^a^		0.001^a^
Non^2^ (*n* = 376)	12131.29 (6441.03, 20971.95)		2162.22 (1272.72, 3724.68)		20.00 (13.00, 28.00)		−424.24 (−2065.42, 51.21)	
Class 0 (*n* = 4)	1755.15 (1063.57, 7935.78)	0.001^a^	426.57 (153.63, 753.75)	0.001^a^	6.50 (3.50, 8.75)	<0.001^a^	−2.45 (−1455.14, 4.48)	0.002^a^
Class I (*n* = 77)	12524.26 (10158.28, 15195.46)		2117.82 (1564.21, 2814.13)		20.00 (15.50, 25.00)		−68.42 (−1868.74, 235.95)	
Class II (*n* = 339)	14308.06 (10312.60, 20076.77)		2393.89 (1762.67, 3525.59)		24.00 (17.00, 35.00)		−1288.12 (−3602.15, 30.45)	
Class III (*n* = 6)	14635.45 (8775.30, 19027.32)		2586.87 (2239.47, 3593.10)		27.00 (18.00, 47.25)		−3947.06 (−5938.21, −1558.56)	

Non^1^: Other types of HAIs that are not related to catheter use. Non^2^: HAIs located at sites other than the surgical site, Class 0: SSI involving a Category 0 incision (no skin incision or laparoscopic incision only). Class I: Superficial incisional SSI. Class II: Deep incisional SSI. Class III: Organ/space SSI.

a *p*-values were estimated using Kruskal–Wallis test.

b *p*-values were estimated using Mann–Whitney U test.

Compared to non-catheter-associated infections, patients with catheter-associated infections [including central line-associated bloodstream infections (CLABSI), ventilator-associated pneumonia (VAP), and catheter-associated urinary tract infections (CAUTI)] exhibited statistically significant differences in Hospitalization costs(*p* < 0.05), but no statistically significant differences in diagnostic tests and procedures, Length of stay or DIP payment differentials (*p* > 0.05) (Table 2).

Significant differences existed among surgical site infections (SSI) by Wound Class and non-SSI in hospitalization costs, diagnostic tests and procedures, length of stay, and DIP payment differentials (*p* < 0.05). Among SSI, greater surgical wound contamination grades were associated with increased hospitalization costs, diagnostic tests and procedures, length of stay, and DIP payment differentials (*p* < 0.05) (Table 2).

### 3.4 Impact of HAIs on medical insurance settlement indicators

After risk-set matching, 379 out of 802 HAI cases were successfully matched with control cases. Hospitalization costs, diagnostic tests and procedures, and length of stay in the infected group were significantly higher than those in the non-infected group (all *p* < 0.05). Crucially, the median DIP payment differentials in the infected group were negative (indicating financial loss), with a statistically significant difference relative to the non-infected group (*p* < 0.05). The Hodges–Lehmann median difference between two groups was $1,436.27, corresponding to an approximate DIP-related loss of $1,436.27 per HAI patient (Table 3).

**Table 3:** Comparison of the medical insurance settlement indicators between the infected and non-infected group.

Indicators	Infected group (n = 379) M (P25, P75)	Non-infected group (n = 379) M (P25, P75)	*Z*	Median difference (95% CI)	*p*-value
Hospitalization costs	13026.96 (8408.80, 18689.41)	7348.88 (5079.90, 11627.60)	10.275	5031.01 (4106.00, 5980.41)	<0.001
Diagnostic tests and procedures	2092.94 (1480.76, 3126.64)	1395.67 (852.71, 2065.96)	9.705	798.81 (640.98, 960.87)	<0.001
Length of stay	20 (14, 29)	12 (8, 16)	12.560	9(7, 10)	<0.001
DIP payment differentials	−435.78 (−2709.55, 46.13)	203.69 (−36.81, 1800.20)	−10.182	−1436.27 (−1794.92, −1119.00)	<0.001

*p*-values were estimated using Mann–Whitney U test. Median differences and 95% CIs were estimated using Hodges–Lehmann estimator.

Results from other matching models were generally consistent with these observations. Notably, risk-set matching produced the smallest Hodges–Lehmann median difference in length of stay between groups, thus minimizing confounding bias related to cost increases driven by prolonged hospitalization. Detailed results are provided in the Supplementary Table S3.

### 3.5 Multivariable analysis of DIP payment differentials

Generalized Linear Model was conducted in the risk set - matched cohort to explore factors independently associated with DIP payment differentials. After adjusting for potential confounders, only the occurrence of HAIs and infection with ≥3 MDRO types were identified as independently associated factors (*p* < 0.05). HAI occurrence and infection with ≥3 MDRO types led to an increase of $2,021.15 and $5,601.44 in DIP deficit, respectively. No other variables showed statistically significant independent associations with DIP payment differentials (Table 4).

**Table 4:** Factors associated with DIP payment differentials after risk-set matching.

Variable	B	Std. error	95% CI	*p*-value
Sex (male as ref)				
Female	−107.18	229.52	(−557.02, 342.67)	0.641
Payment type (non-DIP as ref)				
DIP	8.82	252.15	(−485.39, 503.03)	0.972
Age	−3.98	8.38	(−20.40, 12.44)	0.635
CACI (≤3 as ref)				
>3	−587.31	430.17	(−1430.41, 255.80)	0.172
Invasive device (No as ref)				
Yes	−85.09	260.34	(−595.34, 425.15)	0.744
Surgery (No as ref)				
Yes	−1360.97	1134.63	(−3584.80, 862.86)	0.23
Wound classification (No as ref)				
Class 0	−98.29	383.05	(−849.05, 652.46)	0.797
Class I	1379.93	1142.69	(−859.69, 3619.55)	0.227
Class II	1587.48	1221.04	(−805.71, 3980.67)	0.194
Class III	1082.27	3180.79	(−5151.97, 7316.50)	0.734
HAIs (No as ref)				
Yes	−2021.15	260.87	(−2532.44, −1509.86)	<0.001
Catheter-associated infections (No as ref)				
Yes	149.73	796.76	(−1411.89, 1711.36)	0.851
Number of MDRO infection types (0 as ref)				
1	−527.12	527.81	(−1561.61, 507.36)	0.318
2	−2423.12	1610.15	(−5578.95, 732.71)	0.132
≥3	−5601.44	520.41	(−6621.43, −4581.45)	<0.001

*p*-values were estimated using Generalized Linear Model. Invasive device: urinary catheter, central vascular catheter, and mechanical ventilation. Catheter-associated infections: central line-associated bloodstream infections (CLABSI), ventilator-associated pneumonia (VAP), and catheter-associated urinary tract infections (CAUTI).

## 4 Discussion

To our knowledge, this is the first study to adopt risk-set matching to explore the impact of HAIs on medical insurance settlement indicators within the DIP payment context. The DIP grouping rule is based on “principal diagnosis + primary procedure”, which ensures similar resource consumption across cases in the same group. The scientific validity of this grouping rule has been acknowledged in both practical implementation and academic research (19). In our analysis, gender, age, CACI, and DIP categories were selected as matching covariates for 1:1 matching, propensity score matching, and risk-set matching. A comparison of the three approaches confirmed that risk-set matching effectively mitigates confounding bias and time-dependent bias, which is consistent with previous research findings (18). Furthermore, we applied GLM to the data after risk-set matching, which further adjusted for potential confounders. This design strengthened the accuracy of effect estimation and improved the overall scientific rigor of our conclusions.

Among the top five DIP categories with the highest concentration of HAIs, infected patients showed significantly higher hospitalization costs, diagnostic tests and procedures, and longer length of stay, and DIP payment deficits compared with non-infected patients. However, two major DIP categories exhibited positive DIP payment differentials, indicating a surplus. This suggests that HAIs do not inevitably result in DIP deficits. The underlying reason is that the DIP payment standard is benchmarked based on the average medical expenses of corresponding DIP categories across the city. For an individual hospital, the actual cost for a given category may be either above or below this benchmark. Therefore, for categories where the baseline cost is already lower than the payment standard, even if the total cost increases due to infection, a surplus may still be preserved. This highlights the value of using the DIP payment differential as an indicator to more accurately reflect the actual financial position of hospitals.

In addition, the top five DIP categories were predominantly associated with digestive system malignancies. Taking malignant neoplasm of rectum as an example, compared to laparoscopic anterior resection of rectum alone, the combined procedure with temporary ileostomy exhibited a 1.5-fold higher infection rate (4.42% vs. 6.64%) and a 4.3-fold greater reduction in DIP payment surplus for infected groups ($118.89 vs. $27.66). This indicates that even within the same tumor type, different surgical approaches can directly influence HAI risks and financial outcomes, underscoring the need for disease-specific refined management based on DIP categories. Meanwhile, *Malignant neoplasm of thoracic esophagus: Partial esophagectomy via cervical-thoracic-abdominal three-incision approach* exhibits the largest variation in DIP payment differentials and a relatively high infection cases, and *Malignant neoplasm of cervix: Radical hysterectomy with bilateral salpingo-oophorectomy* incurs the greatest DIP payment loss and a significant prolongation of hospital stay. These findings suggest that hospitals can effectively integrate DIP payment differentials across disease categories into their infection management practices, thereby implementing a targeted, disease-specific key management strategy. Priority should be accorded to DIP categories characterized by both high infection rates and substantial payment deficits. Collaborative efforts involving clinical departments and administrative units should be directed toward identifying infection causes and risk factors, followed by the development of targeted prevention and treatment measures. Such an approach would help reduce infection rates while minimizing additional costs and improving DIP profitability outcomes.

Multiple studies (20–22) have shown that HAIs lead to additional medical expenses, with distinctly varied economic burdens across different infection sites. And different types of infections and the severity of HAIs significantly influence subsequent patient treatment and healthcare expenditures. However, the extent of DIP payment losses associated with tumor-associated infections remains underexplored. In this study, a higher frequency of HAIs during a single hospitalization was positively correlated with progressive increases in hospitalization costs, diagnostic tests and procedures, and length of stay, whereas no significant statistical difference was observed in DIP payment deficits, likely due to the limited number of infection events. In addition, a greater variety of MDRO infections was positively correlated with progressive increases in hospitalization costs, diagnostic tests and procedures, length of stay, and DIP payment deficits. For each additional case of healthcare-associated infection per patient, medical institutions incur an extra loss of approximately $813–$4,152. For each additional MDRO infection acquired, the additional loss amounts to about $546–$3,223. Notably, MDRO infections had the most statistically significant impact on DIP payment differentials, indicating that controlling MDRO infections is crucial for infection prevention and cost containment.

For catheter-related infections, only the hospitalization costs were lower than those for non-catheter infections, while no statistically significant differences were observed in diagnostic test and procedure costs, length of stay, or DIP payment differentials. Current research on catheter-related infections primarily uses non-infected patients as controls (23, 24), with a lack of comparative studies between catheter-related and non-catheter-related infections. Future studies should conduct in-depth analyses from the perspectives of clinical infection characteristics, treatment strategies, and prognosis to explore the reasons for the differences in costs and length of stay between the two groups.

Higher surgical wound contamination grades correlated with increased infection risk and significantly different medical costs across incision types (25, 26). Our study demonstrates that elevated wound contamination levels lead to greater hospitalization costs, diagnostic tests and procedures, longer stays, and larger DIP payment differentials. Notably, median total hospitalization costs, diagnostic tests and procedures, and length of stay for class II/III SSIs were significantly higher than those for patients with other infections (non-SSI). Their DIP payment differentials exceeded twice those of non-SSI patients, highlighting the need to prioritize cost overruns attributable to II/III SSIs.

The core advantages of the DIP payment model include not only reducing hospitalization costs but also appropriately shortening the average length of stay (27). Nevertheless, implementation of this model carries potential risks. As hospitals focus more on cost control, excessive pursuit of medical insurance surpluses may lead to under-provision of medical services, such as premature discharges. Notably, a complex relationship exists between length of stay and infection risk. While prolonged hospitalization may increase HAI risk, early discharge might result in undetected or inadequately treated infections, subsequently elevating mortality and readmission rates (28). Therefore, to reduce time-dependent bias and control for confounding factors, this study applied risk-set matching to accurately estimate the incremental costs and length of stay attributable to HAIs.

After risk-set matching, the analysis showed that HAI patients had significantly higher hospitalization costs, diagnostic test and procedure costs, longer length of stay, and greater DIP deficits compared with the non-infected group. Specifically, HAIs resulted in an increase of $5,031.01 in hospitalization costs, $798.81 in diagnostic test and procedure costs, a 9-day prolongation of length of stay, and additional loss of $1,436.27 in DIP payment differentials. The increase in hospitalization costs was similar to findings reported by Li et al. and higher than those in general hospitals (29, 30), which may be attributed to the greater disease severity among cancer patients and more complex treatment following infection. In contrast, the observed prolongation of hospital stay was shorter than that reported in studies using other matching methods, mainly because risk-set matching effectively reduced time-dependent bias, allowing the results to more accurately reflect the actual impact of HAIs.

GLM analysis showed that both HAIs and MDRO infection were negatively associated with DIP payment differentials. The estimated effect size for HAIs was −$2,021.15. While the impact of MDRO infection intensified with an increasing number of resistant types. Patients infected with three or more drug-resistant types had an effect size of −$5,601.44, substantially exceeding that of HAIs. Notably, the effect size of infection on DIP payment differentials estimated by GLM (−$2,021.15) differed somewhat from the median difference between infected and non-infected groups in the risk-set matching analysis (−$1,436.27). This discrepancy is mainly attributable to the skewed distribution of DIP payment differentials data, with a coefficient of variation of 5.8.

Based on research estimates (31, 32), prevention of all identified HAIs would generate an additional $897 million in total profits, with every ¥1 invested in HAI control averting ¥82 in losses. Given the severity of DIP deficits attributable to HAIs, implementing targeted prevention measures and optimizing the management of high-cost infection types is of critical importance. Under the DIP model, hospitals can integrate HAI incidence and DIP payment differentials across DIP categories to shift infection prevention upstream to the clinical decision-making stage, enabling clinical teams to balance treatment optimization with cost control. Furthermore, HAIs incur substantial deficits and lack corresponding ICD diagnosis codes, making them difficult to capture within DIP categories. When medical insurance funds are sufficient, policymakers may consider adopting a fee-for-service model for such cases during policy formulation, so as to enhance hospitals’ motivation to treat HAIs and ensure that patients with HAIs receive adequate care.

In conclusion, this study systematically evaluated the economic burden of HAIs among cancer patients under China’s DIP payment system. The findings indicate that under the DIP payment model, hospitalization costs no longer accurately reflect a hospital’s real financial balance. In contrast, DIP payment differentials, as a direct measure of actual surplus or deficit, provide a more valid reflection of the economic burden and thus represent a more scientifically robust and managerially meaningful core metric for hospital infection control. Methodologically, the use of risk-set matching helped minimize confounding and time-dependent bias, thereby enhancing the reliability of the effect estimates. At the practical level, a DIP category-specific approach to infection prevention and control offers greater targeting and improved effectiveness, facilitating the dual achievement of healthcare quality and financial performance. This study provides an innovative practice pathway for refined and evidence-based hospital management in the context of DIP payment reform.

As the first DIP-specific study focusing on cancer-associated infections, this study has several limitations. First, although data were obtained from a large tertiary cancer center, the single-center design limits the generalizability of the findings. Variations in medical service pricing, payment models, and DIP classification criteria across regions may affect the external validity of the conclusions. Second, the relatively low incidence of HAIs in this study reflects the well-standardized infection control practice in a specialized cancer hospital and leads to more conservative estimates of economic burden without overestimation bias. However, this also means the findings may not be generalizable to institutions with different levels of infection control or to other types of hospitals. Moreover, the small number of infection events due to the low incidence may reduce the statistical power of subgroup analyses. Finally, this study evaluated the economic burden only from the hospital perspective and did not include data related to patients’ medical insurance reimbursement.

## Data Availability

The original contributions presented in the study are included in the article/Supplementary material, further inquiries can be directed to the corresponding author.
