# Supplementary material for: The economic burden of Hospital-Acquired Infections on cancer patients under China’s pioneering Diagnosis-Intervention Packet payment model: a retrospective study
Source: Front Public Health. 2026 Apr 16;14:1798805. doi: 10.3389/fpubh.2026.1798805 (PMC13128657; doi:10.3389/fpubh.2026.1798805)
Supplement: Supplementary file 1 [file Table_1.docx]

***Supplementary Material***

**Supplementary Table 1.** A modified Charlson Age Comorbidity Index (CACI) Scoring Criteria and Risk Stratification rate

| **Comorbidity** | **ICD-10** | **CACI Baseline Score** |
| --- | --- | --- |
| Myocardial infarction | I21-I22,I25.2 | 1 |
| Congestive heart failure | I50 | 1 |
| Peripheral vascular disease | I70-I73 | 1 |
| Cerebrovascular disease | I60-I69 | 1 |
| Dementia | F00-F03,G30 | 1 |
| Rheumatologic disease | M30-M36、M05.0-M06.9 | 1 |
| Peptic ulcer disease | K25-K28 | 1 |
| Chronic pulmonary disease | J40-J44,J47,J60-J67 | 1 |
| Hemiplegia | G81.0-G81.9 | 2 |
| Renal disease | N18.3-N18.9、N19、Z99.2 | 2 |
| Mild liver disease | B18.0-B18.2、K70.0-K70.1、K71.0、K71.9、K70.9、K73.0-K73.9、K75、K76 | 1 |
| Diabetes without chronic complication | E10.0-E10.1、E10.9、E11.0-E11.1、E11.9、E12.0-E12.1、E12.9、E13.0-E13.1、E13.9、E14.0-E14.1、E14.9 | 1 |
| Diabetes with chronic complication | E10.2-E10.8、E11.2-E11.8、E12.2-E12.8、E13.2-E13.8、E14.2-E14.8 | 2 |
| Moderate to severe liver disease | K70.2-K70.4、K71.1-K71.8、K74.0-K74.6、K72.0-K72.9、Z94.4 | 3 |
| AIDS | B20-B24 | 3 |
| Ages |  |  |
| ＜50 years |  | 0 |
| 50~59 years |  | 1 |
| 60~69 years |  | 2 |
| 70~79 years |  | 3 |
| ≥80 years |  | 4 |

Note: *P* values were estimated using χ² test.

**Supplementary Table 2.** The impact of DIP Settlement Policy on the HAI incidence rate

| **Groups** | **Number of inpatient cases** | **Number of HAIs cases** | **HAI incidence rate（%）** | ***p-*value** |
| --- | --- | --- | --- | --- |
| DIP group | 122725 | 490 | 0.40 | 0.42 |
| non-DIP group | 82844 | 312 | 0.38 |  |

*p-*values were estimated using χ² test.

**Supplementary Table 3.** Comparison of Medical Insurance Settlement Indicators between Infected and Non-infected Groups across Three Models

| **Indicators** | **1:1 matching**  **(n=707/707)** | **conventional propensity score matching**  **(n=802/802)** | **risk set matching**  **(n=379/379)** |
| --- | --- | --- | --- |
| Hospitalization costs |  |  |  |
| Infected group | 12961.75  (8657.90, 18838.38) | 13127.30  (8730.36, 19546.92) | 13026.96  (8408.80, 18689.41) |
| Non-infected group | 6898.56  (4055.92, 10925.93) | 7273.97  (3971.16, 10919.91) | 7348.88  (5079.90, 11627.60) |
| Median difference (95%CI) | 5951.85  (5283.63,6634.85) | 5979.46  (5322.29,6648.63) | 5031.01  (4106.00, 5980.41) |
| *p-*value | ＜0.001 | ＜0.001 | ＜0.001 |
| Diagnostic Tests and Procedures |  |  |  |
| Infected group | 2218.59  (1524.41, 3422.53) | 2284.16  (1541.58, 3504.73) | 2092.94  (1480.76, 3126.64) |
| Non-infected group | 1224.36  (750.46, 1772.93) | 1329.53  (725.91, 1953.36) | 1395.67  (852.71, 2065.96) |
| Median difference (95%CI) | 1039.88  (921.79,1160.85) | 1051.71  (934.23,1172.54) | 798.81  (640.98, 960.87) |
| *p-*value | ＜0.001 | ＜0.001 | ＜0.001 |
| Length of stay |  |  |  |
| Infected group | 21  (15, 30) | 21  (15, 30) | 20  (14,29) |
| Non-infected group | 10  (7, 14) | 11  (7,15) | 12  (8,16) |
| Median difference (95%CI) | 11(10,12) | 11(10,12) | 9(7, 10) |
| *p-*value | ＜0.001 | ＜0.001 | ＜0.001 |
| DIP Payment Differentials |  |  |  |
| Infected group | -628.75  (-2814.80,36.63) | -633.95  (-2830.85,36.83) | -435.78  (-2709.55, 46.13) |
| Non-infected group | 157.77  (0.09,1967.73) | 213.67  (0.16,2010.19) | 203.69  (-36.81, 1800.20) |
| Median difference（95%CI） | -1768.07  (-1990.78,-1516.68) | -1836.31  (-2044.89,-1608.57) | -1436.27  (-1794.92, -1119.00) |
| *p-*value | ＜0.001 | ＜0.001 | ＜0.001 |

*p*-values were estimated using Mann–Whitney U test. Median differences and 95% CIs were estimated using Hodges–Lehmann estimator.
